# DNA Methylation Patterns Facilitate the Identification of MicroRNA Transcription Start Sites: A Brain-Specific Study

**DOI:** 10.1371/journal.pone.0066722

**Published:** 2013-06-24

**Authors:** Tapas Bhadra, Malay Bhattacharyya, Lars Feuerbach, Thomas Lengauer, Sanghamitra Bandyopadhyay

**Affiliations:** 1 Machine Intelligence Unit, Indian Statistical Institute, Kolkata, India; 2 Department of Computer Science and Engineering, University of Kalyani, Kalyani, Nadia, India; 3 Computational Oncology, Eils Labs, German Cancer Research Center, Heidelberg, Germany; 4 Department of Computational Biology and Applied Algorithmics, Max Planck Institute for Informatics, Saarbrücken, Germany; Louisiana State University Health Sciences Center, United States of America

## Abstract

Predicting the transcription start sites (TSSs) of microRNAs (miRNAs) is important for understanding how these small RNA molecules, known to regulate translation and stability of protein-coding genes, are regulated themselves. Previous approaches are primarily based on genetic features, trained on TSSs of protein-coding genes, and have low prediction accuracy. Recently, a support vector machine based technique has been proposed for miRNA TSS prediction that uses known miRNA TSS for training the classifier along with a set of existing and novel CpG island based features. Current progress in epigenetics research has provided genomewide and tissue-specific reports about various phenotypic traits. We hypothesize that incorporating epigenetic characteristics into statistical models may lead to better prediction of primary transcripts of human miRNAs. In this paper, we have tested our hypothesis on brain-specific miRNAs by using epigenetic as well as genetic features to predict the primary transcripts. For this, we have used a sophisticated feature selection technique and a robust classification model. Our prediction model achieves an accuracy of more than 80% and establishes the potential of epigenetic analysis for *in silico* prediction of TSSs.

## Introduction

MicroRNAs (miRNAs) are a class of short (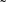
22 nt) non-coding RNAs that control the translation and stability of protein-coding genes [1]. They regulate genes through translational repression or post-transcriptional regulation [2], [3]. Thus, miRNAs are important in many cellular functions and accountable for many diseases [4]. It is known that miRNAs exert regulatory activities in their mature stage, which is reached after cellular processing of primary miRNAs (pri-miRNAs) and precursor miRNAs (pre-miRNAs) transcribed from the DNA. Pri-miRNAs are much longer transcripts that are first transcribed from the DNA. The removal of a portion of pri-miRNA by the nuclear RNase III enzyme Drosha produces the pre-miRNA, a 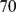
–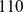 nt intermediate [5]. Finally, the pre-miRNAs become mature miRNAs by the operation of another RNase III enzyme Dicer. The mature miRNAs, along with RISC, bind to the 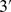 untranslated regions (UTRs) of mRNAs and regulate their expression. A significant amount of information is available about the loci of pre-miRNAs and mature miRNAs. But due to the inadequate information on experimentally validated transcription start sites (TSSs), which manifest the transcription initiation loci of pri-miRNAs, very little is known about pri-miRNA transcripts. The *in silico* prediction of TSSs in the upstream region of pre-miRNAs can contribute significantly to identifying such transcripts. Moreover, recent findings suggest that pri-miRNAs can also take part in the regulation of genes [6]. Therefore, the identification of the pri-miRNA transcripts is of substantial relevance.

In the last few years, the area of prediction of pri-miRNA transcripts has been attracting the attention of researchers [7]–[12]. Understandably, the major focus in this direction is on intragenic miRNAs, i.e. miRNAs located within a gene, as they are co-transcribed with their host genes. Limited work has been conducted for studying the TSSs of intergenic miRNAs, those located between genes. A recent study highlights that miRNA TSSs are different from the TSSs of genes and therefore need specific prediction models [13]. A classification model based on support vector machines (SVM) [14] with a multi-objective optimization based feature selection has been proposed in [13] where known miRNA TSSs are used for training the classifier.

As reported in a current study, intronic, exonic and intergenic regions of DNA exhibit distinct epigenetic characteristics [15]. As of now, only genetic features are considered for TSS identification of miRNAs. But with the development in epigenetics, several new forms of genomewide data have become available. Incorporating features that are based on epigenetic footprints in the DNA appears to be relevant in such studies. There are recent studies in which putative promoters of miRNAs have been identified by analyzing epigenetic features [16]. However, the prediction of exact TSS is a somewhat different problem. In the current analysis, we have collected a large set of genetic and epigenetic features (even though epigenetic footprints in the DNA are also genetic features [17]), some of which are novel, to predict TSSs of human miRNAs. In particular, features based on DNA methylation are employed for the first time for miRNA TSS recognition, to the best of our knowledge. This type of epigenetic modification is of particular relevance, as its influence on promoter regulation has been established before in numerous studies (e.g. reviewed in [18]). Baer *et al.* have recently reported extensive DNA hypermethylation and hypomethylation in miRNA promoters (identified manually) in association with aberrant miRNA expression in chronic lymphocytic leukemia [16]. To facilitate such studies, we have proposed here a machine learning approach to precise TSS identification. Furthermore, in higher vertebrates DNA methylation nearly exclusively appears in the CpG context, where the methylated state of this dinucleotide is the default case [19], [20]. Unmethylated CpGs are often found clustered in so called CpG islands [21], [22], which play an important role in gene regulation. To test whether this relationship also exists for miRNAs, we have included several features based on CpG island characterizations into the analysis [13].

Notably, the epigenetic modifications are tissue-specific [23]. Therefore, the miRNAs expressed in a type of tissue should exhibit distinct epigenetic features. Here, we utilize the available brain-specific methylation data for the prediction of TSSs of miRNAs expressed in the brain. We employ a classifier model based on a Random Forest (RF) [24]. The information on brain tissue-specificity has been collected from available literature. Several recently experimentally validated primary transcripts and associated TSSs have been used for this purpose. Features based on methylation patterns in the genomic region around the TSSs are employed. CpG island based features, in addition to a number of genetic features, are also included [13]. We use a recently proposed feature selection method based on Variable Weighted Maximal Relevance Minimal Redundancy criterion [25]. Finally, the classifier is assessed by cross validation and further tested on independent data.

## Results

First, experiments were conducted to determine whether methylation based information is essential for identifying TSSs of miRNAs expressed in the brain. In the second part of the study, we have analyzed the importance of each of five different categories of features. Next, we have applied the VWMRmR feature selection algorithm and constructed the classification model based on the training dataset with reduced dimensionality. Finally, the performance of the proposed model was compared with those of some other approaches using the prediction results on an independent test dataset.

### Selection of the Best Feature Set

Many genomic regions across the entire genome, that appear to be CpG islands due to repeat elements [22], [26], might increase the number of false positives during promoter prediction. So we study only the non-repetitive part of the sequence, as done in [13], for CpG island determination. Current studies on several organisms show that promoters exhibit specific methylation patterns [15], [27]. Inspired by these, we have conducted an experiment to observe whether the inclusion of methylation-based features improves the classification performance for miRNA TSS prediction or not. For this purpose, we have prepared two types of dataset corresponding to two different feature sets **NM
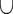
PL
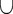
S
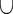
CI** and **NM
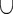
PL
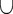
S
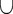
CI
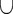
MT**. Each of these datasets has 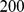 samples 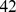 of which correspond to brain-tissue specific TSS samples while 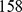 are negative TSS samples. Subsequently, we have trained two separate RF models based on each of the two datasets. The average five-fold cross-validation results, computed over ten independent runs of these two models have been listed in Table 1.

**Table 1:** Performance of the brain-tissue specific miRNA TSS prediction model with and without methylation-based features alongside the other features.

Feature Set	#Features	Classifier Performance		
		Criteria	*μ*	*σ*
		*Acc*	90.65	1.20
		*Sn*	68.10	4.79
**NM∪ PL∪ S∪ CI**	371	*Sp*	96.65	0.60
		*Pr*	84.35	2.74
		*MCC*	0.70	0.04
		*Acc*	91.85	1.31
		*Sn*	70.71	4.77
**NM∪ PL∪ S∪ CI∪ MT**	385	*Sp*	97.47	0.52
		*Pr*	88.07	2.75
		*MCC*	0.74	0.04

The 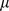 and 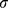 denote mean and standard deviation values of the respective performance metrics.

As can be seen from Table 1, the feature set combination with **MT** provides better results than the other feature set in terms of all of the five evaluation criteria, i.e., accuracy, sensitivity, specificity, precision and 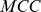. This result demonstrates that inclusion of methylation based features not only improves the prediction capability of the proposed model but also indicates that tissue specific methylation analysis is important.

### Significance Analysis of Features

To assess the importance of the different features including the methylation based features (MT) introduced in the present study, the F-scores [28] are computed. If the number of positive and negative samples are 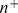 and 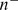, respectively, then the F-score of the 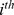 feature is computed as
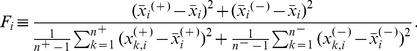
(1)


Here, 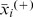, 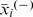 and 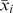 stand for the mean values of the 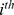 feature over the set of the entire positive samples, the entire negative samples and total samples, respectively. Again, 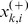 denotes the 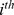 feature of the 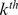 positive sample and 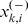 represents the 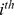 feature of the 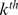 negative sample. A larger value of F-score is an indicator of a more discriminative feature. All 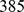 features were ranked based on their F-scores, where a larger value gains a lower (better) rank. The summary of the F-score analysis for different feature subsets is shown in Table 2. In this table, rankwise importance of all the five aforementioned feature sets is displayed separately. Additionally, the NM feature set is partitioned into five different subsets, namely, NM-CG (all possible n-mer containing CG as a substring), NM-1 (for 1-mers), NM-2 (for 2-mers), NM-3 (for 3-mers) and NM-4 (for 4-mers). As can be seen from Table 2, the class of special features (S) comprises better discriminators (as highlighted by the ranks) of the TSS pattern than the other classes. Note that, only three features out of the total 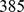 belong to the category of special features (S). Even though these few features may not be sufficient by themselves to identify brain-specific miRNA TSS, this analysis underlines the importance of their inclusion. It is also evident from the table that all CpG island based features have been ranked within the top 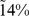 in the total ranked list. This observation once again confirms that they are very useful for TSS prediction of miRNAs [13]. Furthermore the average rank of the 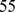 NM-CG features is 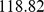 which is less than half of the average rank found using NM. This signifies that NM-CG is also an effective feature set. In fact, recent reports highlight that epigenetic marks also depend upon DNA sequences [17].

**Table 2:** Analysis of the importance of features by F-score.

Summary	Feature Type									
Statistics	NM	NM-CG	NM-1	NM-2	NM-3	NM-4	CI	PL	S	MT
Minimum Rank	3	7	16	7	3	11	4	130	1	94
Maximum Rank	385	373	77	352	382	385	53	253	43	300
Average Rank	207.7	118.82	51.5	155.69	165.33	228.61	26.92	185.5	15.33	160.93

A major drawback of the F-score is that the mutual information among different features is ignored [28]. To overcome this deficit, we have applied VWMRmR on the full feature set, which produces a sorted ranked list of the 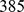 features. The summary of the analysis of feature importance for the same ten feature subsets (as was shown in Table 2) is provided in Table 3. Similar to the analysis of features importance by F-score, this table also confirms that features in the “S” category need to be included in the miRNA TSS feature set. This analysis confirms that CI is a good feature subset. Additionally, almost the same observation is found about NM-CG like the F-score analysis. The methylation features appear to gain in importance as compared to the F-score analysis. Indeed, in the top 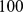 features, now there are 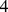 MT features compared to only 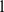 feature appeared in the F-score analysis. Also for the VWMRmR the best rank for a methylation feature is obtained at position 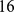, whereas for F-score this value was 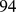.

**Table 3:** Analysis of the importance of features by VWMRmR feature selection.

Summary	Feature Type									
Statistics	NM	NM-CG	NM-1	NM-2	NM-3	NM-4	CI	PL	S	MT
Minimum Rank	2	2	55	7	10	2	4	212	1	16
Maximum Rank	385	280	372	380	385	381	124	301	9	341
Average Rank	206.06	110.48	188.25	214.25	228.48	203.49	44	270.25	4.33	149.64

### Performance Evaluation on an Independent MiRNA TSS Dataset

There are several gene TSS prediction tools developed to date [29]–[31]. Almost all are based on machine learning approaches by using TSS samples of protein-coding genes. However, the recent investigations suggest that miRNA TSSs can be improved by applying miRNA-specific training datasets [13]. Therefore we have tested our model, incorporating tissue specificity and methylation features, on an independent test set.

The performances of three existing gene TSS prediction algorithms were compared with that of our proposed brain-specific miRNA TSS prediction model on an independent miRNA TSS dataset described in the *Materials* section. The first method, CoreBoost_HM, is a recently developed RNA polymerase II core-promoter prediction tool that is entirely dedicated to the human genome [29]. In this tool, explicit features based on genome-wide histone modification are incorporated together with features relating to DNA sequence. The second tool, Dragon TSS Desert Masker (DDM), is a well-known gene TSS prediction tool that not only recognizes large segments of mammalian genomes as non-TSS locations (NTL) but also identifies true TSSs with high accuracy [30]. This research also reveals that approximately above 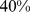 of the human genome are most likely NTLs. The classification results employing the DDM tool are obtained by setting the sensitivity level (approx. percentage of real TSSs not masked) to medium (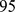
%). The last tool, Easy Promoter Prediction Program (EP3), is a core promoter prediction model developed using large-scale structural features of DNA [31]. In this tool, the default window size of 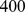 is used for obtaining the classification results.

The comparative performance of the methods has been assessed in terms of five evaluation criteria, namely, accuracy, sensitivity, specificity, precision and 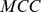 using that test dataset. The classification results of these four prediction models are listed in Table 4. It can be observed from the table that the proposed prediction model outperforms all other prediction tools in terms of three evaluation criteria, i.e., accuracy, sensitivity and MCC. The accuracy, sensitivity, specificity, precision and 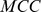 of the proposed model are 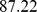
%, 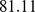
%, 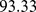
%, 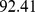
% and 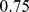, respectively. Although the specificity and the precision obtained using CoreBoost_HM are higher than those found using our miRNA TSS model, its sensitivity value ( = 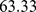
%) is extremely low as compared to that of our model. In comparison with DDM, the proposed model provides better results in each of the aforementioned five evaluation criteria. Although the specificity and precision obtained with EP3 are higher than those of the proposed approach, the prediction power of EP3 recognizing true TSSs is very poor. The proposed model is the only one that achieves greater than 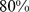 sensitivity as well as specificity. To summarize, incorporation of methylation data is found to be effective in predicting TSSs of miRNAs expressed in the brain.

**Table 4:** Comparison of the performance of three existing gene TSS prediction algorithms along with our proposed method in predicting brain-tissue specific miRNA TSS.

	Training	Classifier Performance based on the Features				
Algorithm	Sample Type	*Acc*	*Sn*	*Sp*	*Pr*	*MCC*
**CoreBoost_HM**	Gene TSSs	80.86	63.33	97.78	96.61	0.65
**DDM**	Gene TSSs	81.67	74.44	88.89	87.01	0.64
**EP3**	Gene TSSs	72.78	45.56	100	100	0.54
**Proposed**	miRNA TSSs	87.22	81.11	93.33	92.41	0.75

Best mean values of the percentage accuracy, sensitivity, specificity, precision and 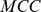 are shown in bold.

## Discussion

The present article deals with the problem of predicting TSSs of miRNAs by incorporating several novel epigenetic features along with the other existing relevant sequence based features. The study on brain-specific miRNAs since the methylation data is available for brain tissue. A sophisticated RF classification model has been constructed using a brain-specific miRNA TSS dataset. The positive samples in this miRNA TSS dataset were collected from a recent miRNA TSS database designed using high-throughput sequencing data. We have evaluated the prediction capability of the brain-specific TSS prediction model using an independent miRNA dataset. The performance of this model is compared to those of some other existing machine learning based gene TSS prediction models. The computational results demonstrate that the proposed model performs very well as compared to existing methods being the only one that provides both a sensitivity and specificity above 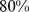.

In the future, we plan to include additional epigenetic features like histone modification and activation of small non-coding RNAs. We are also trying to collect additional positive samples in order to assemble a well-balanced brain-specific miRNA TSS training dataset. Studies on other tissues is another important direction of future work.

## Materials

A set of brain specific miRNAs was collected by a literature survey. Then, the reported TSSs were divided into training and test sets as described below. Furthermore, the feature set used is described in detail.

### Sample Collection

We have carried out extensive literature survey to collect more than eighty brain-specific miRNAs (see Text S1 for more details). We have extracted the positive TSS samples corresponding to these miRNAs and further prepared an effective negative set for training the TSS prediction model. We have also accumulated a separate set of TSS samples for further testing purposes. The methylation data is obtained from MethylomeDB [23] which reports genomewide methylation patterns based on the hg18 genome assembly. We have mapped all the data resources used in this study to the hg18 genome build.

A few recent studies attempted to experimentally verify the TSSs of miRNAs. A detailed review on this can be found in [32]. Chien *et al.* were the first to apply high-throughput sequencing to identifying miRNA TSS [12]. They provide exact TSS information, rather than a region, for 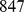 human miRNAs. From this large set of miRNAs, 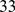 human miRNAs, which correspond to 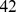 different TSS loci, are identified as brain-specific based on our literature survey (see Text S1). The methylation map we used is given in hg18 at a single base resolution. So, we have converted the others. Since the TSS information has been mapped to the hg19 genome build, we have further mapped it to the hg18 version using the Lift-Over tool of GALAXY [33]. We extract a 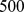 bp stretch of genomic sequence, that includes 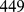 bp upstream and 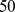 bp downstream region around each miRNA TSS, from the UCSC genome browser (NCBI36/hg18 genome build) [34]. All these 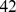 brain-specific samples comprise positive training data for the prediction model. To our knowledge, no benchmark set with negative samples for brain-tissue specific miRNA TSS is available in the literature. In recent papers, the importance of adding negative samples for making a robust biological prediction model has been highlighted. For the TSS prediction problem, we have selected 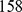 negative samples (in the form of 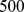 bp sequence) randomly from the entire genome in such a way that no known miRNA lies within a region of 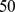 kb either upstream or downstream of the corresponding sample loci, as no TSS is likely to be found at a locus that is within 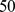 kb of the 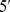 end of the corresponding miRNA [11]). In this way, a total of 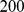 samples (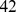 positive samples and 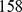 negative samples) have been collected as the training data. Several existing and novel features have been extracted from these TSS samples, as described later in this section, to comprise the final training dataset.

We prepared an independent set of test data for validating the performance of the classifier. For this purpose, we have used the information provided in Marson *et al.* [9]. They report several miRNA TSSs defined over a stretch of 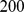 bp or more. The data for only the brain-specific miRNAs are considered here. A region around the center of the 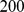 bp stretch is taken as a positive TSS sample. Ninety such positive samples have been collected. Ninety negative samples have also been collected as described earlier. This provides a set of 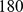 independent test samples.

### Description of Features

For the prediction of brain-tissue specific miRNA TSS, a large number of features has been generated based on diverse sequence characteristics as well as epigenetic properties. Some of these were used in [13], while some are new. These can be grouped into five different categories as follows:

#### 1. N-mer Features (NM)

The frequencies for 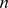-mers (for 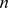 = 1, 2, 3 and 4) are collected from a sequence by considering only its valid subsequence segment. A valid subsequence is represented as a portion of a given sequence that contains only the four bases ‘A’, ‘T’, ‘G’ and ‘C’. In contrast, an ‘N’ is used to denote an undefined base. As the 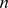-mer based features are taken from diverse samples, they are normalized by dividing with the length of the corresponding valid sequence segment. In this way, a total 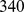 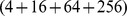 features are obtained.

#### 2. Palindromic Features (PL)

The occurrence of several palindromic subsequences with half length 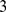, 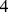, 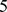 and 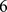 are extracted from the valid portion of the given sample sequence. Similar to 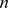-mer features generation, their frequencies are normalized by dividing each of them by the length of the corresponding valid sequence portion. In this manner, a total of 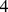 features are collected.

#### 3. Special Features (S)

We include three over-represented special subsequence patterns that are frequent in promoters [35]. The different forms of these three patterns are: G**G, G**G**G and GC**GC**GC in which the wildcard character ‘*’ represents either one of A/T/C/G. Analogously to the above two feature categories, these three features are also normalized.

#### 4. CpG Island Based Features (CI)

According to Gardiner-Garden *et al.*, a genomic region that contains higher density of G+C and CpG than average in the whole genome is called a CpG islands [21]. A large fraction of human promoters comprises high CpG content [36]. Some studies related to CpG islands emphasizes that unmethylated CpGs are frequently found in clusters inside the CpG islands [21], [22]. This cluster formation plays a significant role for determining the patterns of gene regulation. Usually, CpG islands are characterized by two feature values, the value of CpG O/E (CpG observed over expected ratio) and G+C content (cumulative occurrence of C and G). These values are calculated along the sequence with a sliding window approach. Determining a suitable window length is a challenging job. In a recent study of Hackenberg *et al.*, the problem of choosing the *ad hoc* value for the length of examined region has been addressed [37]. A number of CpG-related studies highlight that CpG-islands can be better characterized by considering only the non-repetitive portion of the sequence rather than the entire sequence [13]. This is possibly because many regions that comprise repeat elements (like Alu repeats), which are abundant in the genome, resemble CpG islands [22], [26]. Therefore many false-positive regions may come into view as CpG-rich promoters. Inspired by this observation, both the CpG O/E and G+C pair values are computed from the non-repeated portion of the given region of interest. These values can be calculated either with overlapping or non-overlapping sliding windows. Inspired from an earlier observation [13], we have considered non-overlapping windows of lengths {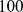 bp, 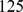 bp, 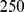 bp and 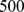 bp} over the entire region of interest. The CpG O/E value is calculated as 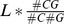, where 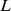 denotes the length of the non-repeated sequence analyzed. On the other hand, G+C content is calculated as 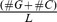. In this way, a total 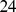 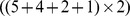 features have been defined.

#### 5. Methylation Based Features (MT)

DNA methylation is a common epigenetic modification of cytosines in CpG dinucleotides. Unmethylated CpGs cluster in CpG islands. We use the recently published database MethylomeDB [23] to compute MT features for the positive and negative samples of 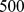
bp regions. This database offers genome-wide DNA methylation profiles corresponding to brain-tissue of both human and mouse. There are a total of 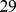 human brain samples corresponding to three different cortical regions, namely, dorsolateral prefrontal cortex (dlPFC), ventral prefrontal cortex (vPFC) and auditory cortex (AC). Among these 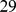 samples, 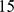 (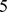 dlPFC, 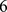 vPFC and 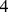 AC) are schizophrenia disease samples whereas 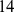 (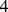 dlPFC, 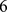 vPFC and 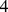 AC) are non-psychiatric controls. For the present research work, we have analyzed only the methylation patterns from non-psychiatric controls. For each of the specified regions, the methylation score is computed based on the methylated sites falling within that region. Let 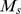 be the probability of a site (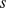) being methylated, within the region under consideration, and 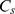 be the sequence read coverage. Then, the feature value is computed as
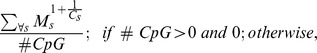
where 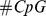 denotes the count of CpG islands in the region studied. The rationale behind this normalized score is to give importance to higher methylation probability and penalizing it for lower read coverage (see Text S1 for more details). In this way, total 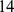 MT features are generated, one for each of the 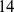 non-psychiatric control samples.

## Methods

The feature selection algorithm, the RF based classification model and the brain-tissue specific TSS prediction models are described in the following subsections.

### Feature Selection Algorithm

For many real-life applications, feature selection is necessary because a lot of the features are irrelevant or redundant [38]. Feature selection algorithm differ in the strategy employed for searching for feature subsets and in the score that measures the importance of a feature subset. Mutual information is widely used in feature selection algorithms due to its ability to identify non-linear dependence between two features. Mutual information between two random variables measures the mutual dependence between the two variables. The Variable Weighted Maximal Relevance Minimal Redundancy criterion based feature selection (VWMRmR) [25] is a recently proposed algorithm that utilizes an existing normalized variant of mutual information [39] to compute both the class relevance as well as the average redundancy of the candidate feature. Earlier approaches like the Maximal Relevance Minimal Redundancy criterion based feature selection algorithm (mRMR) [40], Normalized Mutual Information based Feature Selection (NMIFS) [41] and Improved Normalized Mutual Information based Feature Selection (INMIFS) [42], considered the weight of class relevance and the average redundancy equally, and these two weights have been retained throughout the steps of feature selection. The VWMRmR approach is a weighted version of the mRMR method in which the weight of the average redundancy is continuously increased with respect to the number of features that have already been selected while a fixed weight value is set for the class relevance. The performance of the VWMRmR has been evaluated to be superior to several other existing mutual information based feature selection algorithms, namely, maximal relevance based feature selection (MR), mRMR and INMIFS, based on analyses of six real-life high dimensional datasets. In this article we have selected the topmost 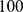 features according to VWMRmR.

### The RF based Classification Model

An RF has been trained for the purpose of building a classification model. The WEKA software [43] has been used for this purpose. There are two important parameters that need to be set, i.e., numFeatures (the number of features to be employed in each random selection) and numTrees (the number of decision trees to be produced). For the purpose of validation, we have set both of these values to 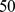 based upon sensitivity analysis. The performance of the corresponding RF model has been assessed using five-fold cross validation and this was repeated five times to obtain a single mean estimate. Five evaluation criteria, namely accuracy (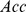), sensitivity (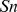), specificity(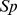), precision(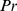) and Matthews correlation coefficient (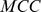), are used. These are defined as follows:
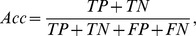


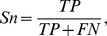


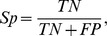


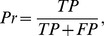


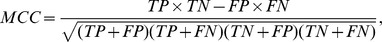
where 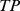, 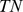, 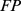 and 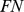 denote the number of true positives, true negatives, false positives and false negatives, respectively.

### Proposed Brain-tissue Specific MiRNA TSS Prediction Model

From the training data, a set of 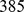 features was extracted, as described earlier. Then the VWMRmR algorithm [25] was applied to select the top 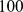 features. These were used to train a RF-based classifier as already described. This model was used for a brain-tissue specific miRNA TSS prediction. Here we have posed the problem of TSS identification as a binary classification problem. The capability of this model was assessed using an independent testing data as described in the Results section.

## Supporting Information

Text S1 **Details about the collection of brain-specific miRNAs, preparation of miRNA TSS dataset, and the construction of the methylation-based feature score.**
(PDF)
