# Correction: DNA Methylation Patterns Facilitate the Identification of MicroRNA Transcription Start Sites: A Brain-Specific Study

**DOI:** 10.1371/annotation/dd8f4acc-3859-46e2-9136-20b6b4d08d21

**Published:** 2013-12-30

**Authors:** Tapas Bhadra, Malay Bhattacharyya, Lars Feuerbach, Thomas Lengauer, Sanghamitra Bandyopadhyay

Several errors were introduced in the preparation of this article for publication.

In Table 1, the first two columns have been combined. Please see the corrected Table 1 here: 

**Figure:**
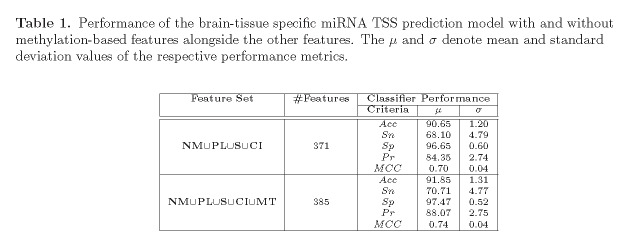


In the PDF version of this manuscript, the ~ symbol in the first line of the Introduction has been miniaturized. The sentence should read: "MicroRNAs (miRNAs) are a class of short (~22 nt) non-coding RNAs that control the translation and stability of protein-coding genes."

In the fourth to last sentence of the "Significance Analysis of Features" portion of the Results section, the ~ symbol has been misplaced above 14%. The sentence should read: "It is also evident from the table that all CpG island based features have been ranked within the top ~14% in the total ranked list." 

