# Supplementary material for: DNA Methylation Patterns Facilitate the Identification of MicroRNA Transcription Start Sites: A Brain-Specific Study
Source: PLoS One. 2013 Jun 24;8(6):e66722. doi: 10.1371/journal.pone.0066722 (PMC3691241; doi:10.1371/journal.pone.0066722)
Supplement: Text S1 — Details about the collection of brain-specific miRNAs, preparation of miRNA TSS dataset, and the construction of the methylation-based feature score. (PDF) [file pone.0066722.s001.pdf]

DNA Methylation Patterns Facilitate the Identification of MicroRNA Transcription Start Sites: A Brain-specific Study (Supplementary Details)

Tapas Bhadra[1], Malay Bhattacharyya[2], Lars Feuerbach[3], Thomas Lengauer[4], Sanghamitra Bandyopadhyay[1,*]
1 Machine Intelligence Unit, Indian Statistical Institute, 203 B. T. Road, Kolkata – 700108, India
2 Department of Computer Science and Engineering, University of Kalyani, Kalyani, Nadia – 741235, India
3 Computational Oncology, Eils Labs, German Cancer Research Center, Im Neuenheimer Feld 580, 69120 Heidelberg, Germany
4 Department of Computational Biology and Applied Algorithmics, Max Planck Institute for Informatics, Saarbrücken, Germany
∗ **E-mail: sanghami@isical.ac.in**

Introduction

In this supplement, we describe about the collection of brain-specific microRNAs (miRNAs) and the preparation of miRNA TSS dataset (to feed the classifier) in more detail. Furthermore, we justify the construction of the methylation-based feature score used in the analysis.

Collection of Brain-specific MiRNAs

The miRNAs associated with brain-specific diseases should be active in the brain but may not in other tissues, i.e. we expect them to be brain tissue-specific. Based upon this hypothesis, we collect the miRNAs known to have relation with the Alzheimer's disease and Schizophrenia, two important diseases originating from brain. From Human MicroRNA Disease Database (HMDD) [1], we obtain such disease associated miRNAs (see Tables 1-2). We have further carried out extensive literature survey to find out the miRNAs that have been experimentally verified as brain tissue-specific. These miRNAs are listed in Table 3. All the reports about these miRNAs are enclosed with the publication link in PubMed. These three records together (reported in the Tables 1-3) provide 82 brain specific miRNAs.

MiRNA Name	PubMed Link
hsa-miR-101-1	http://www.ncbi.nlm.nih.gov/pubmed/21172309
hsa-miR-103-1/2	http://www.ncbi.nlm.nih.gov/pubmed/21179570
hsa-miR-106b	http://www.ncbi.nlm.nih.gov/pubmed/19110058
hsa-miR-107	http://www.ncbi.nlm.nih.gov/pubmed/18234899
hsa-miR-128-1/2	http://www.ncbi.nlm.nih.gov/pubmed/21686130
hsa-miR-137	http://www.ncbi.nlm.nih.gov/pubmed/21994399
hsa-miR-146a	http://www.ncbi.nlm.nih.gov/pubmed/18801740
hsa-miR-17	http://www.ncbi.nlm.nih.gov/pubmed/19110058
hsa-miR-181c	http://www.ncbi.nlm.nih.gov/pubmed/21994399
hsa-miR-20a	http://www.ncbi.nlm.nih.gov/pubmed/19110058
hsa-miR-21	http://www.ncbi.nlm.nih.gov/pubmed/19683563
hsa-miR-29a	http://www.ncbi.nlm.nih.gov/pubmed/20202123
hsa-miR-29b-1	http://www.ncbi.nlm.nih.gov/pubmed/18434550
hsa-miR-34c	http://www.ncbi.nlm.nih.gov/pubmed/21946562
hsa-miR-590	http://www.ncbi.nlm.nih.gov/pubmed/21548758
hsa-miR-9-1/2/3	http://www.ncbi.nlm.nih.gov/pubmed/21994399

Table 1. MiRNAs reported in HMDD to have association with the Alzheimer's disease.

MiRNA Name	PubMed Link
hsa-let-7g	http://www.ncbi.nlm.nih.gov/pubmed/18184693
hsa-miR-106b	http://www.ncbi.nlm.nih.gov/pubmed/17326821
hsa-miR-137	http://www.ncbi.nlm.nih.gov/pubmed/21926974
hsa-miR-15a	http://www.ncbi.nlm.nih.gov/pubmed/19721432
hsa-miR-15b	http://www.ncbi.nlm.nih.gov/pubmed/19721432
hsa-miR-195	http://www.ncbi.nlm.nih.gov/pubmed/17326821
hsa-miR-198	http://www.ncbi.nlm.nih.gov/pubmed/17849003
hsa-miR-206	http://www.ncbi.nlm.nih.gov/pubmed/17849003
hsa-miR-20b	http://www.ncbi.nlm.nih.gov/pubmed/17326821
hsa-miR-212	http://www.ncbi.nlm.nih.gov/pubmed/17326821
hsa-miR-24-1/2	http://www.ncbi.nlm.nih.gov/pubmed/17326821
hsa-miR-26b	http://www.ncbi.nlm.nih.gov/pubmed/17326821
hsa-miR-29a	http://www.ncbi.nlm.nih.gov/pubmed/17326821
hsa-miR-29b-1	http://www.ncbi.nlm.nih.gov/pubmed/17326821
hsa-miR-29c	http://www.ncbi.nlm.nih.gov/pubmed/17326821
hsa-miR-30a	http://www.ncbi.nlm.nih.gov/pubmed/17326821
hsa-miR-30b	http://www.ncbi.nlm.nih.gov/pubmed/17326821
hsa-miR-30d	http://www.ncbi.nlm.nih.gov/pubmed/17326821
hsa-miR-30e	http://www.ncbi.nlm.nih.gov/pubmed/17326821
hsa-miR-346	http://www.ncbi.nlm.nih.gov/pubmed/19264453
hsa-miR-34a	http://www.ncbi.nlm.nih.gov/pubmed/21738743
hsa-miR-7-1/2/3	http://www.ncbi.nlm.nih.gov/pubmed/17326821
hsa-miR-9-1/2/3	http://www.ncbi.nlm.nih.gov/pubmed/17326821
hsa-miR-92a-1/2	http://www.ncbi.nlm.nih.gov/pubmed/17326821
hsa-miR-92b	http://www.ncbi.nlm.nih.gov/pubmed/17326821

Table 2. MiRNAs reported in HMDD to have association with Schizophrenia.

TSS Sample Collection for Brain-specific MiRNAs

A handful of recent studies tried to experimentally verify the TSSs of miRNAs [2]. In general, these existing resources, providing information on miRNA TSS, report the region of interest that might include the original TSS. In some cases, they report multiple TSS regions corresponding to a single miRNA. Based on the 82 brain-specific miRNAs we identified, we found brain-specific miRNA TSSs (available as regions or loci) of diverse counts (57, 10, 90, 22, 17 and 42 TSSs, respectively from the sources [3–8]). The major problem here is that the amount of requisite information is little for many sources. By using this small number of positive samples, a good classification model that can perform well on some previously unseen samples is not easy to build. Thus the limited information available from [4, 6, 7] was ignored. Most of the other studies provide the miRNA TSS information in terms of a genomic stretch of a certain length, even though these lengths exceed 1 million for some miRNAs. As Chein *et al.* report a reasonably good number of miRNA TSSs, experimentally identified as a single genomic locus, we have utilized their research output in the present study. The brain-specific miRNA TSSs collected for training the classifier are shown in Table 4.

Methylation-Based Feature Score

Suppose, M_s and C_s denote the probability of observing the methylated state and the read coverage (number of observations) of any arbitrary site s, respectively. Then, the methylation-based feature value for the site s, say $F(s)$, is expected to be proportional to M_s, i.e.

$$F_s \propto M_s.$$

Now, in order to properly adjust the methylation probability in cases of lower read coverage, we define M_s in such a way that it decreases with decreasing C_s. Therefore, we use the value

$$F_s = M_s^{1+\frac{1}{C_s}}.$$

MiRNA Name	PubMed Link
hsa-let-7	http://www.ncbi.nlm.nih.gov/pubmed/18485210
hsa-miR-1	http://www.ncbi.nlm.nih.gov/pubmed/12007417
hsa-miR-101	http://www.ncbi.nlm.nih.gov/pubmed/18485210
hsa-miR-122a	http://www.ncbi.nlm.nih.gov/pubmed/17019647
hsa-miR-124	http://www.ncbi.nlm.nih.gov/pubmed/21957233
hsa-miR-124a	http://www.ncbi.nlm.nih.gov/pubmed/18485210
hsa-miR-125	http://www.ncbi.nlm.nih.gov/pubmed/18485210
hsa-miR-1271	http://www.ncbi.nlm.nih.gov/pubmed/20864449
hsa-miR-128	http://www.ncbi.nlm.nih.gov/pubmed/21596314
hsa-miR-128b	http://www.ncbi.nlm.nih.gov/pubmed/21841775
hsa-miR-129	http://www.ncbi.nlm.nih.gov/pubmed/21157891
hsa-miR-132	http://www.ncbi.nlm.nih.gov/pubmed/19958814
hsa-miR-133	http://www.ncbi.nlm.nih.gov/pubmed/17019647
hsa-miR-134	http://www.ncbi.nlm.nih.gov/pubmed/16421561
hsa-miR-135	http://www.ncbi.nlm.nih.gov/pubmed/15003116
hsa-miR-136	http://www.ncbi.nlm.nih.gov/pubmed/12007417
hsa-miR-137	http://www.ncbi.nlm.nih.gov/pubmed/22068596
hsa-miR-140	http://www.ncbi.nlm.nih.gov/pubmed/18485210
hsa-miR-142-5p	http://www.ncbi.nlm.nih.gov/pubmed/21157891
hsa-miR-146	http://www.ncbi.nlm.nih.gov/pubmed/17019647
hsa-miR-15	http://www.ncbi.nlm.nih.gov/pubmed/18485210
hsa-miR-153	http://www.ncbi.nlm.nih.gov/pubmed/18485210
hsa-miR-183	http://www.ncbi.nlm.nih.gov/pubmed/15003116
hsa-miR-184	http://www.ncbi.nlm.nih.gov/pubmed/18203756
hsa-miR-190	http://www.ncbi.nlm.nih.gov/pubmed/15003116
hsa-miR-196	http://www.ncbi.nlm.nih.gov/pubmed/18485210
hsa-miR-206	http://www.ncbi.nlm.nih.gov/pubmed/12007417
hsa-miR-21	http://www.ncbi.nlm.nih.gov/pubmed/17965831
hsa-miR-219	http://www.ncbi.nlm.nih.gov/pubmed/15003116
hsa-miR-222	http://www.ncbi.nlm.nih.gov/pubmed/19351827
hsa-miR-24	http://www.ncbi.nlm.nih.gov/pubmed/17019647
hsa-miR-25	http://www.ncbi.nlm.nih.gov/pubmed/21157891
hsa-miR-26	http://www.ncbi.nlm.nih.gov/pubmed/15003116
hsa-miR-27	http://www.ncbi.nlm.nih.gov/pubmed/18485210
hsa-miR-29	http://www.ncbi.nlm.nih.gov/pubmed/18485210
hsa-miR-30	http://www.ncbi.nlm.nih.gov/pubmed/18485210
hsa-miR-31	http://www.ncbi.nlm.nih.gov/pubmed/18485210
hsa-miR-326	http://www.ncbi.nlm.nih.gov/pubmed/19955368
hsa-miR-331	http://www.ncbi.nlm.nih.gov/pubmed/18485210
hsa-miR-338	http://www.ncbi.nlm.nih.gov/pubmed/19020050
hsa-miR-34	http://www.ncbi.nlm.nih.gov/pubmed/17019647
hsa-miR-34a	http://www.ncbi.nlm.nih.gov/pubmed/20190569
hsa-miR-365	http://www.ncbi.nlm.nih.gov/pubmed/17019647
hsa-miR-375	http://www.ncbi.nlm.nih.gov/pubmed/18485210
hsa-miR-378	http://www.ncbi.nlm.nih.gov/pubmed/18485210
hsa-miR-448	http://www.ncbi.nlm.nih.gov/pubmed/18485210
hsa-miR-503	http://www.ncbi.nlm.nih.gov/pubmed/18485210
hsa-miR-541	http://www.ncbi.nlm.nih.gov/pubmed/21452340
hsa-miR-9/9*	http://www.ncbi.nlm.nih.gov/pubmed/18624795
hsa-miR-92b	http://www.ncbi.nlm.nih.gov/pubmed/18624795
hsa-miR-96	http://www.ncbi.nlm.nih.gov/pubmed/20864449

Table 3. The miRNAs having brain specificity as reported in various literature.

Here, the lower bound of the methylation probability ($M_s \leq 1$) is M_s^2, where $C_s = 1$. Note that the feature value approaches M_s with as the read coverage increases to infinity. To further remove the bias of CpG islands, we set the normalized feature value to

$$\frac{\sum_{\forall s} M_s^{1+\frac{1}{C_s}}}{\#CpG}.$$

miRNA Name	Type	Chromosome	Strand	TSS	TSS (Experiment)
hsa-mir-101-1	intergenic	1	-	65524191	65532194
hsa-mir-103-1	intragenic	5	+	167987909	167956376
hsa-mir-103-2	intragenic	20	-	3898210	3906693
hsa-mir-124-1	intragenic	8	-	9760982	9776596
hsa-mir-124-2	intragenic	8	+	65291706	65285788
hsa-mir-124-3	intragenic	20	+	61809852	61808955
hsa-mir-124-3	intragenic	20	+	61809852	61808955
hsa-mir-129-1	intragenic	7	+	127847925	127846868
hsa-mir-129-2	intragenic	11	+	43602944	43600647
hsa-mir-132	intergenic	17	-	1953674	1960152
hsa-mir-134	intergenic	14	+	101488403	101438989
hsa-mir-136	intergenic	14	+	101335397	101314819
hsa-mir-142	intergenic	17	-	56408679	56409879
hsa-mir-146a	intragenic	5	+	159901409	159895244
hsa-mir-181c	intergenic	19	+	13985513	13976434
hsa-mir-183	intergenic	7	-	129414854	129420061
hsa-mir-184	intergenic	15	+	79502130	79463212
hsa-mir-206	intergenic	6	+	52009147	52001071
hsa-mir-20b	intergenic	X	-	133304308	133307920
hsa-mir-21	intergenic	17	+	57918627	57915327
hsa-mir-212	intergenic	17	-	1953674	1960152
hsa-mir-219-1	intergenic	6	+	33175612	33171308
hsa-mir-219-2	intergenic	9	-	131154993	131198003
hsa-mir-222	intergenic	X	-	45606530	45624430
hsa-mir-29a	intragenic	7	-	130562298	130596999
hsa-mir-29b-1	intragenic	7	-	130562298	130596999
hsa-mir-29b-2	intergenic	1	-	207975868	207997156
hsa-mir-29c	intergenic	1	-	207975868	207997156
hsa-mir-30b	intergenic	8	-	135817188	135844546
hsa-mir-30d	intergenic	8	-	135817188	135844546
hsa-mir-331	intergenic	12	+	95702196	95692643
hsa-mir-34a	intergenic	1	-	9211836	9259025
hsa-mir-34c	intragenic	11	+	111383663	111383204
hsa-mir-365-1	intragenic	16	+	14397824	14396078
hsa-mir-365-1	intragenic	16	+	14397824	14396078
hsa-mir-365-1	intragenic	16	+	14397824	14396078
hsa-mir-375	intragenic	2	-	219866430	219866698
hsa-mir-378	intragenic	3	+	10371913	10343170
hsa-mir-541	intergenic	14	+	101488403	101438989
hsa-mir-92a-2	intergenic	X	-	133304308	133307920
hsa-mir-92b	intergenic	1	+	155164968	155164787
hsa-mir-96	intergenic	7	-	129414854	129420061

Table 4. Information about 42 brain-specific miRNAs (in hg19 genome assembly) collected from [8].

To appropriately handle the cases where CpG islands might be completely absent, we define the feature value as

$$\frac{\sum_{\forall s} M_s^{1+\frac{1}{C_s}}}{\#CpG};\ \text{if}\ \#CpG > 0\ \text{and}\ 0;\ \text{otherwise},$$

where $\#CpG$ denotes the count of CpG islands in the region under inspection.

References

1. Lu M, Zhang Q, Deng M, Miao J, Guo Y, et al. (2008) An analysis of human microRNA and disease associations. PLoS One 3: e3420.

2. Bhattacharyya M, Das M, Bandyopadhyay S (2012) miRT: A database of validated transcription start sites of human microRNAs. Genomics Proteomics Bioinformatics 10: 310-316.

3. Landgraf P, Rusu M, Sheridan R, Sewer A, Iovino N, et al. (2007) A mammalian microRNA expression atlas based on small RNA library sequencing. Cell 129: 1401-1414.

4. Saini HK, Griffiths-Jones S, Enright AJ (2007) Genomic analysis of human microRNA transcripts. Proc Natl Acad Sci U S A 104: 17719-17724.

5. Marson A, Levine SS, Cole MF, Frampton GM, Brambrink T, et al. (2008) Connecting microRNA genes to the core transcriptional regulatory circuitry of embryonic stem cells. Cell 134: 521-533.

6. Fujita S, Iba H (2008) Putative promoter regions of miRNA genes involved in evolutionarily conserved regulatory systems among vertebrates. Bioinformatics 24: 303-308.

7. Corcoran DL, Pandit KV, Gordon B, Bhattacharjee A, Kaminski N, et al. (2009) Features of mammalian microRNA promoters emerge from polymerase II chromatin immunoprecipitation data. PLoS One 4: e5279.

8. Chien CH, Sun YM, Chang WC, Chiang-Hsieh PY, Lee TY, et al. (2011) Identifying transcriptional start sites of human microRNAs based on high-throughput sequencing data. Nucleic Acids Res 39: 9345-9356.
